# Life history evolution and cellular mechanisms associated with increased size in high‐altitude *Drosophila*

**DOI:** 10.1002/ece3.2327

**Published:** 2016-07-25

**Authors:** Justin B. Lack, Amir Yassin, Quentin D. Sprengelmeyer, Evan J. Johanning, Jean R. David, John E. Pool

**Affiliations:** ^1^ Laboratory of GeneticsUniversity of Wisconsin‐Madison425‐G Henry MallMadisonWisconsin53706; ^2^ Laboratoire Evolution, Génomes, Comportement, Ecologie (EGCE)CNRS, Univ. Paris‐Sud, IRDUniversité Paris‐Saclay1 av. de la Terrasse91198Gif‐sur‐YvetteFrance; ^3^ Present address: Center for Cancer Research National Cancer InstituteNIH BethesdaMaryland20892‐1201

**Keywords:** Cell, *Drosophila*, egg, life history, size, wing

## Abstract

Understanding the physiological and genetic basis of growth and body size variation has wide‐ranging implications, from cancer and metabolic disease to the genetics of complex traits. We examined the evolution of body and wing size in high‐altitude *Drosophila melanogaster* from Ethiopia, flies with larger size than any previously known population. Specifically, we sought to identify life history characteristics and cellular mechanisms that may have facilitated size evolution. We found that the large‐bodied Ethiopian flies laid significantly fewer but larger eggs relative to lowland, smaller‐bodied Zambian flies. The highland flies were found to achieve larger size in a similar developmental period, potentially aided by a reproductive strategy favoring greater provisioning of fewer offspring. At the cellular level, cell proliferation was a strong contributor to wing size evolution, but both thorax and wing size increases involved important changes in cell size. Nuclear size measurements were consistent with elevated somatic ploidy as an important mechanism of body size evolution. We discuss the significance of these results for the genetic basis of evolutionary changes in body and wing size in Ethiopian *D. melanogaster*.

## Introduction

Size is a conspicuous characteristic in essentially any organism. In spite of copious amounts of natural variation within and among species, the complexity of size and growth determination has made it incredibly challenging to characterize the genetic basis of evolutionary shifts in body or appendage size (Nijhout et al. 2014). Size is a complex trait at the genetic level, and many hundreds of genes may affect growth in *Drosophila melanogaster* (Turner et al. 2011). The developmental complexity of size – different structures and tissues grow at different rates, begin and end growth at different times, involving an interplay between cell growth, proliferation, and death – can also make size variation extremely difficult to study in even the most tractable laboratory models, let alone in naturally occurring populations of organisms. Furthermore, the size of both whole organisms and individual structures can be highly dependent upon environmental variables (i.e., nutritional input and developmental temperature) (Robertson 1960; French et al. 1998), making quantitative comparisons of size somewhat context dependent, and further complicating its study.

Because *Drosophila* and many other insects grow specifically as larvae, one obvious strategy for evolving larger size is to extend larval development. Consistent with this hypothesis, extended larval development has been observed following artificial selection for larger size in *D. melanogaster* (Partridge and Fowler 1993), while selection for shorter development time yielded smaller flies (Nunney 1996). In other words, a trade‐off could exist between development time and adult size.

In *Drosophila* and other ectotherms, the evolution of increased size at high altitude or latitude is often observed and could reflect adaptation to development at reduced temperatures (Atkinson 1994; Partridge et al. 1994a; Reeve et al. 2000). In addition, fitness experiments have indicated that larger body size conveys a fitness advantage at reduced developmental temperatures (Reeve et al. 2000), but the specific mechanism remains unknown. One explanation suggests that increased competition for larval food resources at warmer temperatures favors rapid development, while reduced competition in cooler environments favors longer development and therefore increased adult size (Partridge and French 1996). Concordantly, development time increases with altitude and latitude for some *D. melanogaster* populations and other insects (Norry et al. 2001; Sambucetti et al. 2006; Folguera et al. 2008), but not in all studied cases (Louis et al. 1982; James and Partridge 1995; Blanckenhorn and Demont 2004; Hodkinson 2005; Collinge et al. 2006). In Australian *D. melanogaster*, haplotypes of the *Neurofibromin* gene were correlated with antagonistic effects on development time and wing size (Lee et al. 2013).

Beyond development time, another life history trait that may interact with adult size is the degree of maternal investment in each egg. Across many insects, larger eggs tend to produce larger adults, while egg size and egg number are inversely correlated (Parsons 1962; Harvey 1983, 1985; Berrigan 1991; but see Fischer et al. 2005; Hassall et al. 2006), potentially reflecting variation along the continuum between r and K reproductive strategies in terms of offspring number versus offspring size. Likewise in *D. melanogaster*, artificial selection for larger eggs reduces female fecundity (Schwarzkopf et al. 1999). Larger egg size within this species has been associated with larger hatching larvae and faster developmental rate, but not with adult body size (Azevedo et al. 1996, 1997), and it has been found to result from laboratory evolution at low temperature (Azevedo et al. 1997).

At the cellular level, size variation can occur in two ways: modification of cell number and/or cell size. For mammalian systems, size variation within and between species is generally driven by cell number (McMahon and Bonner 1983), with the exception of a few specific cell types and tissues (e.g., erythrocytes, megakaryocytes, and trophoblasts) (Promislow 1991; Zybina and Zybina 1996; Zimmet and Ravid 2000). In insects (and many other organisms), cell size changes may arise more readily due to the prevalence of the endocycle: most terminally differentiated tissues have elevated ploidy, because DNA replication and cell growth happen without cell division (Smith and Orr‐Weaver 1991; Edgar and Orr‐Weaver 2001). This mechanism has previously been implicated in driving body and appendage size variation (Scholes et al. 2013). For *Drosophila*, cell size and number have both been implicated in driving size variation, but their relative importance in evolution not fully clear. In the context of developmental plasticity, wing size variation from developmental temperature experiments is entirely driven by cell size (Partridge and French 1996). However, in an evolutionary context, heritable variation in wing size is most often a result primarily of cell number variation, but cell size has been implicated as well. For Hawaiian *Drosophila* species, cell size explained between one‐third and two‐thirds of organ and body size variation (Stevenson et al. 1995). In *D. subobscura*, variation in wing size in North America was primarily a result of cell size variation, but resulted from cell number variation in South America (Calboli et al. 2003). For *D. melanogaster*, wing size variation among most investigated populations is primarily the result of cell number variation (James et al. 1995; Zwaan et al. 2000; Klepsatel et al. 2014), although in South America, size variation resulted from equal contributions of cell number and area (Zwaan et al. 2000), and artificial evolution experiments under cold conditions resulted in the evolution of increased wing size due entirely to increases in cell size (Partridge et al. 1994b). Further data points would be helpful in assessing the relative importance of these mechanisms, especially in terms of the cellular basis of evolutionary change in nature.

Here, we investigate body and wing size phenotypes in *D. melanogaster* from a recently discovered high‐elevation Ethiopian population (3070 m above sea level), which are the largest known naturally occurring *D. melanogaster* in the world. We set out to test two hypotheses regarding the developmental and cellular mechanisms of size evolution. First, we tested whether size might result from extended larval development. But instead, we found that large Ethiopian flies arose from larger eggs, but produced fewer of them, suggesting an alternative life history strategy at high altitude. Second, we tested whether tissue size evolution resulted from changes in cell proliferation or cell size. We discovered an important role for cell size in shaping thoracic muscle and wing size, implying that somatic polyploidy helps drive these size phenotypes, but the evolution of wing size appeared to be mainly driven by cell proliferation.

## Materials and Methods

### Fly populations and stocks

**Figure 1:**
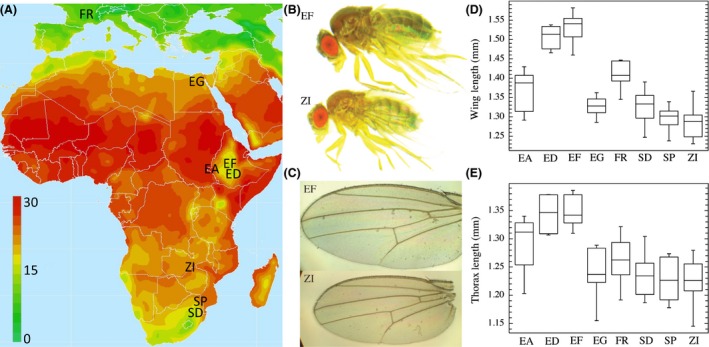
The enlarged wing and body size of highland Ethiopian populations (ED/EF) is shown in comparison with other African populations and one European population (France FR). (A) Sample locations are superimposed upon a map of average temperature (°C). Population information is given in Table A1. Map is courtesy of The Nelson Institute Center for Sustainability and the Global Environment, University of Wisconsin‐Madison. (B and C) Representative body and wing size images from a high altitude Ethiopian population (EF) and an ancestral range, lowland, Zambian population. (D) Box‐plot representation of wing length variation for the eight populations examined. (E) Boxplot representation of thorax length variation for the eight population examined. Specific wing and thorax length measurements are illustrated in Fig. A1. Box plots: horizontal line within box, median; box, lower and upper quartiles; capped vertical lines, 95% confidence.

Phenotypic analyses utilized inbred lines derived from wild‐caught isofemales, collected from eight populations (seven African, one European) that varied in altitude and latitude (Table A1, Fig. 1). These included population samples from Egypt (EG from Cairo), Ethiopia (EA from Gambella, ED from Dodola, and EF from Fiche), France (FR from Lyon), South Africa (SD from Dullstroom and SP from Phalaborwa), and Zambia (ZI from Siavonga). Within populations, individual lines were founded by a single wild‐caught female and then inbred through at least eight generations of full sib matings. Due to the potential for inbreeding and/or long‐term maintenance in the laboratory to affect fitness and trait variance (Lynch and Walsh 1998), all phenotypic measurements (with the exception of the phenotypic plasticity analysis; described below) and experiments were conducted using F1 flies produced from crossing virgin females from one line to males of another line from the same population (with all replicates within each population resulting from completely unique crosses), therefore minimizing the influence of rare deleterious alleles. Unless noted otherwise, all crosses and fly rearing occurred in a single incubator at 20°C (a temperature that occurs commonly in all sample locations), 70% humidity, and a 12:12 h light:dark cycle. Flies were raised at 25°C on medium prepared in batches of 4.5 L water, 500 mL cornmeal, 500 mL molasses, 200 mL yeast, 54 g agar, 20 mL propionic acid, and 45 mL tegosept 10% (in 95% ethanol). Because larval density can significantly affect wing and body size (Imasheva and Bubliy 2003), crosses were conducted in half pint bottles with 20 virgin females and 20 males allowed to oviposit for 48 h.

### Phenotypic measurements

We expand on the thorax and wing size data reported by Lack et al. (2016) by including additional populations and by assessing additional traits. We report thorax and wing length data from six sub‐Saharan African populations, as well as single populations from Europe and northern Africa (Table A1). In addition, we quantified temperature‐driven plasticity in wing and thorax length by examining high‐ and low‐altitude lines from a range of developmental temperatures. To begin to understand the cellular mechanisms underlying variation in these phenotypes, we examined wing and thoracic muscle cell size variation in the phenotypic outlier populations from the Ethiopian highlands as well as from an “ancestral range” Zambian population (Pool et al. 2012). We also compared egg‐laying rate, egg size, and development time between a larger‐bodied Ethiopian population and the smaller‐bodied Zambian population.

**Figure A1:**
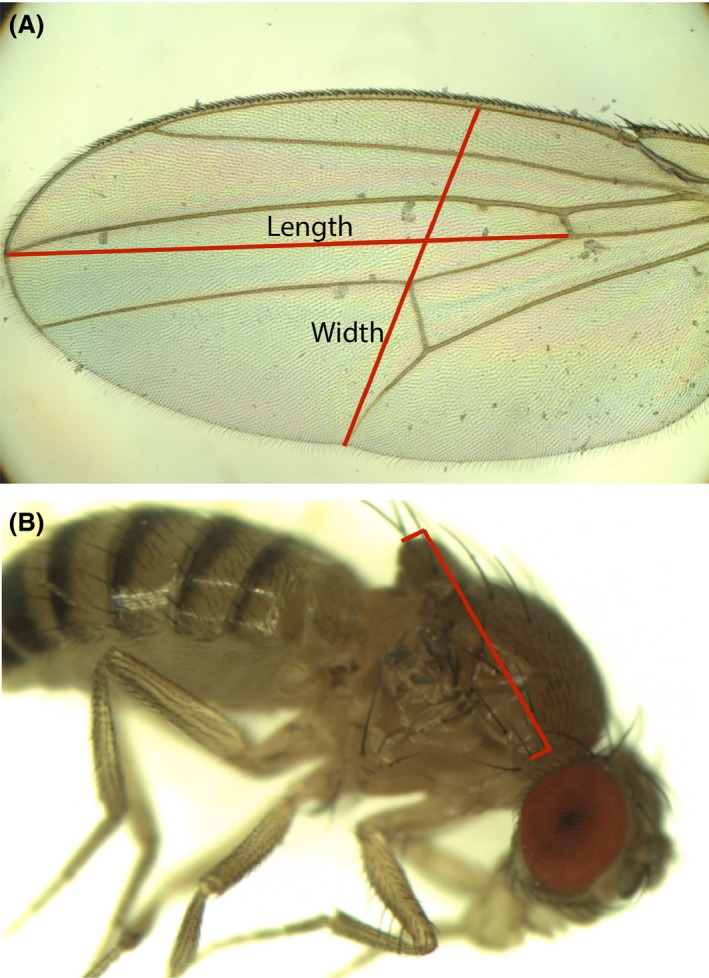
Illustration of the wing (A) and thorax size (B) measurements.

As a proxy of overall body size, we quantified thorax length in 3‐ to 5–day‐old F1 adult females raised as described above. For each independent cross, 10–20 females were photographed with a digital camera attached to a stereo dissecting microscope (AmScope SM‐4BX), and thorax was measured from the base of the anterior humeral bristle to the posterior tip of the scutellum. For wing size, we similarly examined 3‐ to 5‐day‐old F1 adult females from crosses generated as described above. For five females per cross, a wing was removed and photographed at 50× magnification using a digital camera attached to a compound microscope (Olympus BH‐2). The length and depth of each wing were then measured using ImageJ version 1.48 (http://imagej.nih.gov/ij/), and the specific measurements are illustrated in Figure A1. For the length measurement, we measured a straight line drawn from the intersection of the anterior crossvein and L4 longitudinal vein, to where the L3 longitudinal vein intersects the wing margin. For depth, we measured a straight line from the intersection of the L5 longitudinal vein and the posterior wing margin, passing through the intersection of the posterior crossvein and L4, and terminating at the anterior wing margin. To determine wing loading, we estimated the per‐fly mass and wing area for five independent crosses each from the highland Ethiopian population and the lowland Zambian populations. We estimated mass by weighing batches of 20 5‐day‐old adult F1 females to an accuracy of ±0.01 mg. For wing area, we imaged individual wings using the “wing grabber” apparatus described by Houle et al. (2003), and wing area was determined by outlining each wing using ImageJ version 1.48 (http://imagej.nih.gov/ij/), and the reported area for each cross is the mean of the five wings.

In addition to comparisons at a single developmental temperature, we also examined wing and thorax length at a range of temperatures for a single isofemale line from the high‐altitude Ethiopian population (EF98) and a single isofemale line from the lowland Zambian population (ZI18). Rather than facilitating an in‐depth analysis of plasticity and reaction norms, this comparison served to establish that the observed phenotypic divergence between high‐ and low‐altitude populations was not a product of developmental temperature. For this experiment, separate groups of 10 females were allowed to oviposit in vials for 12 h at each of the following temperatures: 14, 17, 21, 25, 28, and 31°C. Phenotypic measurements for this objective were the mean of 10 adult females, and with traits defined as follows. For wing length, measurements were conducted from the point of articulation between the wing and thorax, to the distal tip of the wing, where the L3 vein intersects the wing margin. For thorax length, measurements were conducted from the point of articulation between the head and thorax to the posterior tip of the scutellum.

To examine whether observed body size differences may be resulting from differences in the duration and/or rate of growth, we assayed development time for the highland EF population and the lowland Zambian population. For this assay, we examined five unique crosses from each population (EF and ZI). For each cross, 12 virgin females and 12 males were placed onto grape juice medium and allowed to mate and oviposit for 4 h at 20°C on the bench top. All eggs were then transferred to vials containing standard yeast–cornmeal–molasses fly medium. To keep humidity constant between the benchtop and pressure chamber crosses, a hole was drilled in the bottom of each vial, which was then placed in a small petri dish containing 150 mL of water, 7.5 mL of a 10 *μ*g/mL tetracycline solution, and 1.5 mL 20% tegosept antifungal solution. Vials were then checked every four hours for adult flies, and development time (from the onset of egg‐laying until the eclosion of each adult) was measured from the time point the first fly was seen for each vial until the last adult emerged.

In addition to growth rate variation, we also assayed egg‐laying rate and egg size in the EF and ZI populations to determine whether female reproductive strategy may be contributing to size variation. To measure egg‐laying rate, the same crosses and conditions were utilized as in the development time assay. Twelve virgin females and 12 males were placed onto grape juice medium and allowed to mate and oviposit for 24 h, and then, eggs were counted. This same approach was used to estimate egg size for F1s resulting from 10 different crosses for each of the high‐altitude Ethiopian population (EF) and the ancestral Zambian population (ZI). For each of the unique crosses, five eggs were photographed with a digital camera attached to a stereo dissecting microscope (AmScope SM‐4BX, Irvine, CA), and each egg was traced using ImageJ version 1.48 (http://imagej.nih.gov/ij/) to estimate egg circumference. The five circumference measurements were then averaged to estimate the egg size for each cross.

To ensure that population differences observed above (or lack thereof) were not the result of atmospheric pressure environment, we also examined development time and egg‐laying rate at an air pressure of 524 mmHg, which simulates the approximate air pressure at 3000 m elevation. All phenotypic assays were conducted exactly as described above, with the exception of allowing the parental flies to lay eggs inside a pressure chamber connected to a digital vacuum regulator (J‐KEM Scientific, Model 200, University City, MO) maintaining a constant pressure of 524 mmHg, and then maintaining the offspring in the chamber until assaying for egg number and development time.

### Cell size assays

To examine the relative contributions of cell size and cell area to the wing and thorax size variation among populations, we quantified cell size in the wing epithelium and the dorsal longitudinal muscles (DLMs) of adult females, as well as nucleus size in the DLMs. All cellular measurements were performed on F1 flies from crosses between independent pairs of inbred strains from the same population sample. These flies were kept at controlled temperature (20°C) and humidity (70%) throughout development, and larval density was controlled by placing 20 virgin females with 20 males in bottles, and allowing females to oviposit for 48 h.

**Figure A2:**
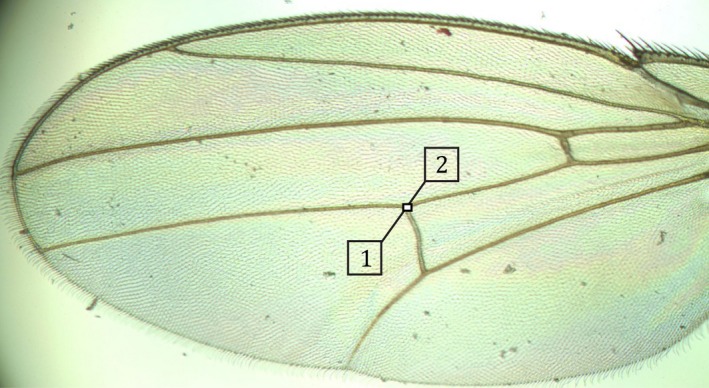
Illustration of the location of the two 0.03‐mm^2^ boxes within which individual trichomes were counted.

For wing cell size, we removed a wing from 3‐ to 5‐day‐old adult females and it was photographed at 100× magnification using a digital camera attached to a compound microscope (Olympus, BH‐2). Cell density was then measured in two standard wing regions (shown in Fig. A2) by counting trichomes in a 0.03‐mm^2^ box.

For skeletal muscle measurements, we focused on the DLMs of the adult female thorax. Flies were raised in the same manner as the wing cell size analysis, and 3‐ to 5‐day‐old adult females were pinned on the dorsal surface of the abdomen with 0.10‐mm minutiens (Austerlitz Insect Pins (R)) in a Sylgard (R) 184 silicone elastomer plate filled with phosphate‐buffered saline (PBS) at pH 7.4 (Life technologies 10010‐023, Carlsbad, CA). A razor blade was used to make an incision on the dorsal side of the thorax to allow fixatives to reach the thoracic muscles. PBS was then replaced with 4% formaldehyde (BDH0500) in PBS, and flies were fixed overnight at 4°C. Flies were then washed twice with PBS and the thorax was bisected, cleaned from debris, and placed in blocking solution consisting of 0.1% Triton X‐100 (Invitrogen HFH10, Carlsbad, CA) and 0.1% normal goat serum (Invitrogen 500622) in PBS for 1 h. Bisected thoraces were then mounted on microscopic slides in Vectashield (R) mounting medium with DAPI (Vector Laboratories Inc. H‐1200, Burlingame, CA).

For each mounted, bisected thorax, we conducted three measurements (illustrated in Fig. 3). To quantify whole muscle size, we measured the width of the third DLM at the center of the thorax at 100× magnification using a digital camera attached to a compound microscope (Olympus, BH‐2, Tokyo, Japan). To approximate muscle fiber size and nucleus size, we then obtained confocal images of DAPI‐stained thoraces on a microscope (LSM 510; Carl Zeiss, Oberkochen, Germany) with a 63× oil objective and accompanying software. Muscle fiber width was measured for eight crosses from each of the EF and ZI populations, three individuals per cross, and five arbitrarily chosen fibers (within a single muscle) per individual. For nucleus size, a minimum of 10 nuclei from the third DLM were traced using ImageJ version 1.48, and replicate measurements for each line were averaged to estimate nucleus circumference. For all skeletal muscle measurements, simple *t*‐tests were used to test for significant differences between the EF and ZI populations.

## Results

### Body and wing size

Expanding on the results presented by Lack et al. (2016), our size data are consistent with previous studies (*e.g.,* David and Capy 1988) showing that temperate populations of *D. melanogaster* tend to be larger than tropical populations (*e.g.,* FR vs. ZI in Fig. 1). However, we found that highland Ethiopian populations (ED and EF; Fig. 1) had still larger thorax and wing lengths than temperate samples and are likely to represent the largest described natural isolates of *D. melanogaster*. In our analysis, thorax length was larger in general within Ethiopia, but was especially large for the two high‐altitude populations (EF and ED; Fig. 1), with these two populations distinct from all others.

**Figure A3:**
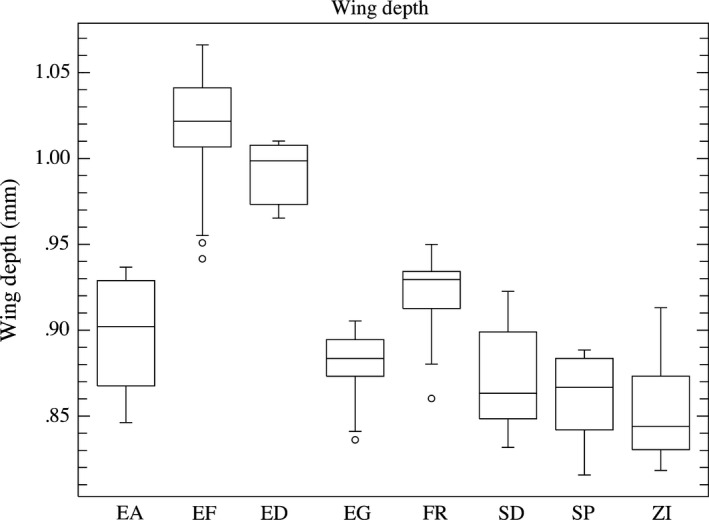
Box plot representation of variation in wing width among the eight analyzed populations included here. Population abbreviations are defined in Table A1, and the specific measurement is illustrated in Figure A1.

**Figure A4:**
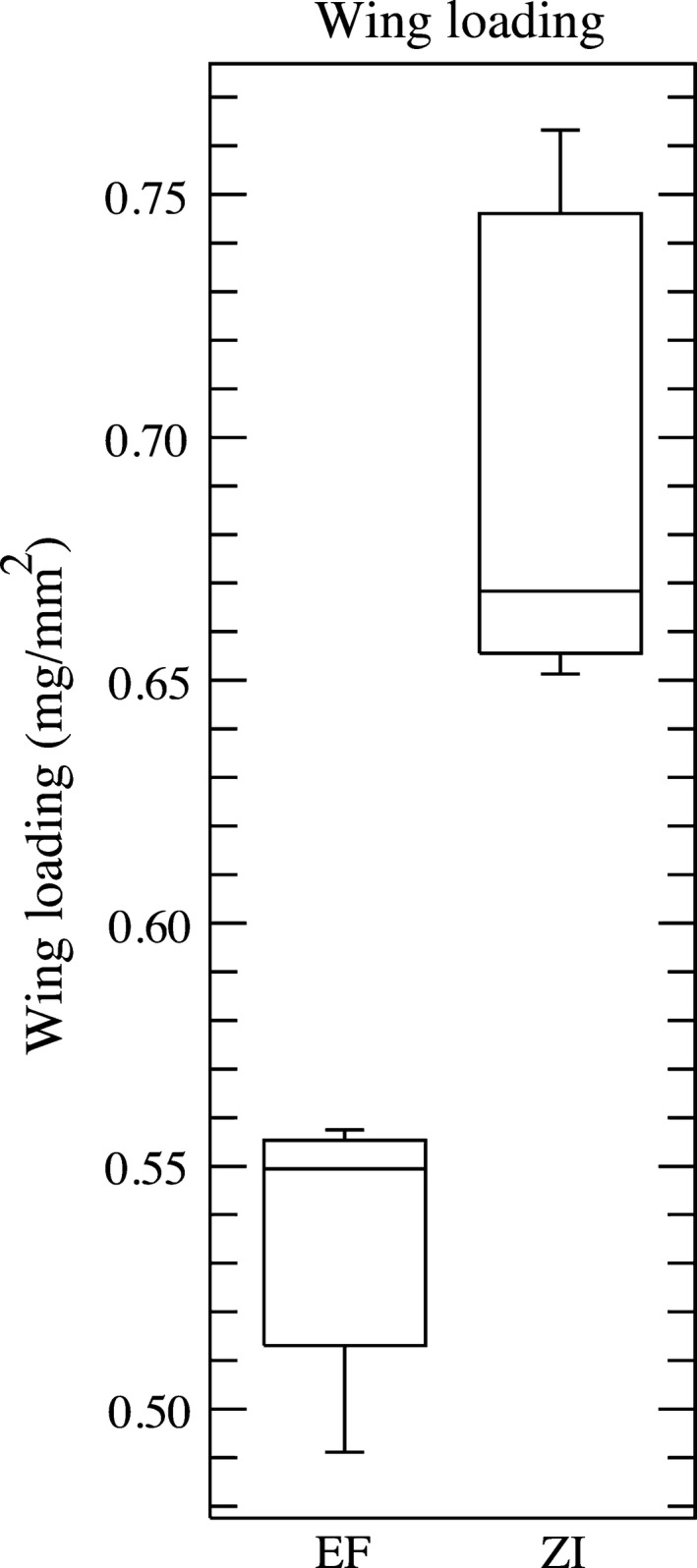
Box plot representation of variation in female wing loading (mass/wing area) between the highland Ethiopian population (EF) and the lowland Zambian populations (ZI). Values for mass, wing area, and loading for each population are given in Table A3. Box plots: horizontal line within box, median; box, lower and upper quartiles; capped vertical lines, 95% confidence interval.

Wing size was also much larger in the highland Ethiopian flies relative to any other sampled *D. melanogaster*, and this was true for both wing length and depth (Figs. 1 and A3, respectively). The wing size increase is proportionately larger than that of the body size increase, as can be seen by the significant reduction in mean wing loading (Fig. A4, Table A3; *t *=* *6.3404, df = 8, *P *=* *0.0002) observed for highland Ethiopia when compared to lowland Zambia (EF = 0.00054 mg/mm^2^; ZI = 0.00069 mg/mm^2^). The greater size increase of wings could reflect wing‐specific evolution or allometric change. Size evolution does appear to be a product of directional selection on size or correlated traits; as shown by Lack et al. (2016), wing size differentiation between EF and ZI greatly exceeds genomewide patterns of genetic differentiation, suggesting that wing size has not evolved neutrally.

We also confirmed that these size differences were qualitatively maintained independent of developmental temperature. While the above data were collected at 20°C, the increased thorax and wing size of EF relative to ZI was maintained over a range of developmental temperatures (Table A4), even as both populations showed the expected plastic gain in size with decreasing temperature. Upon confirming this consistent size difference between the ancestral range ZI population and the phenotypically derived EF population, we continued to focus on this representative population comparison as we investigated size‐related life history traits and cellular mechanisms of growth.

### Development time, egg‐laying rate, and egg size

**Figure 2:**
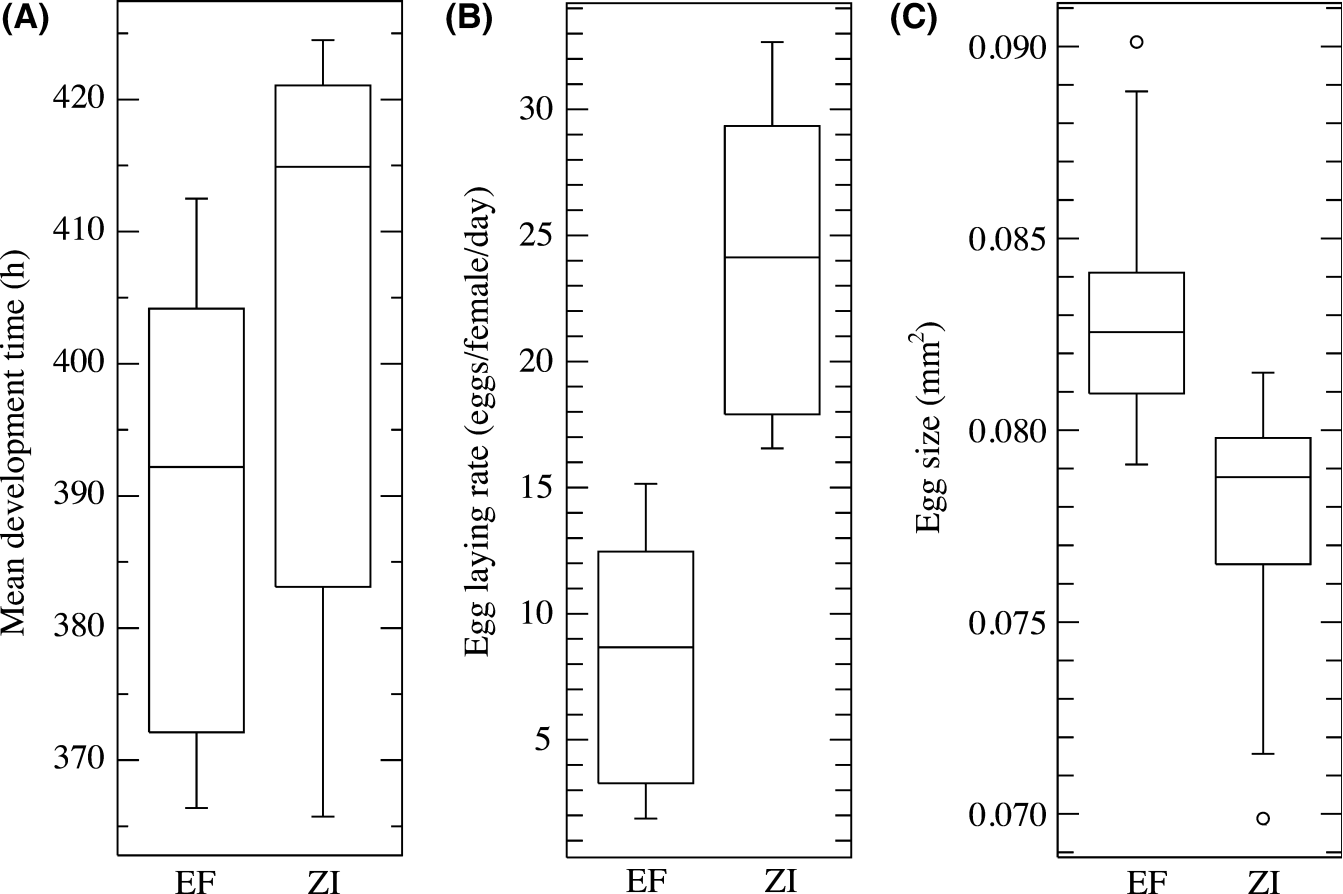
Ethiopian flies develop as quickly as Zambian flies, but lay fewer and larger eggs. Box plots depict variation in development time (A), egg‐laying rate (B), and egg size (C) between a highland Ethiopian population (EF) and the ancestral range, lowland, Zambian population (ZI). Box plots are as in Figure 1.

**Figure A5:**
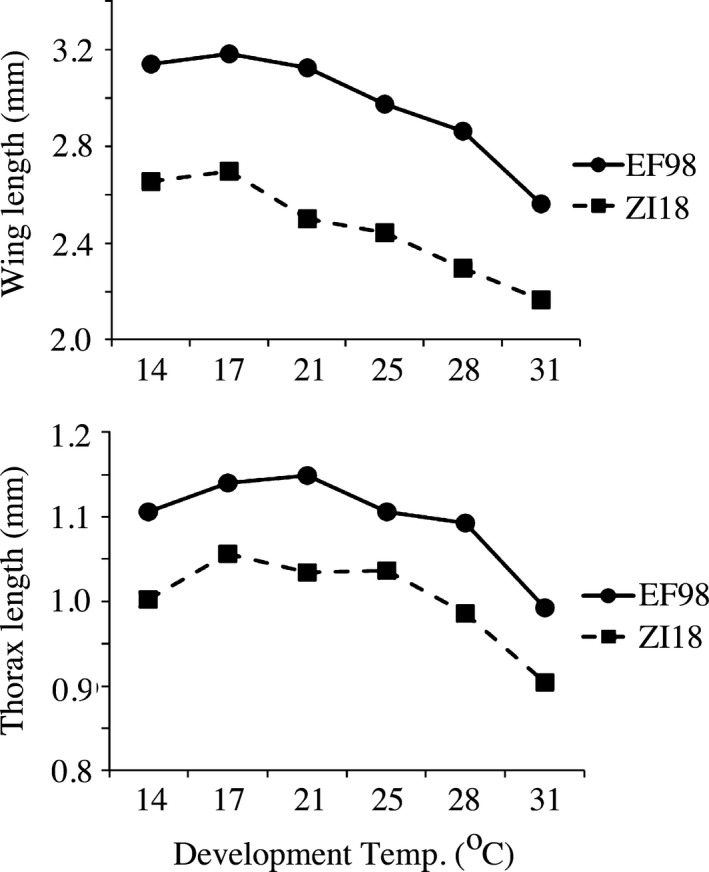
Large wing length (top panel) and thorax length (bottom panel) of Ethiopian flies are consistent across developmental temperatures. Figures show average lengths (across ten females) for the high‐altitude Ethiopian line EF98 and the low‐altitude Zambian line (ZI18) at a range of developmental temperatures. Values are given in Table A4.

One clear hypothesis for a developmental mechanism underlying the evolution of large adult body size is an increased larval development time, allowing for increased mass prior to pupation. However, we detected no statistically significant difference in development time between EF and ZI (Fig. 2; *t *=* *1.0563, df = 8, *P *=* *0.3217), with EF actually having a slightly faster mean time to emergence than ZI (EF = 388.9 h, SD = 19.18; ZI = 398.1, SD = 21.47). We also tested whether this relationship was maintained in the low air pressure environment of the EF population's native high altitude. Here again, no difference was detected between EF and ZI (Fig. A5; Table A5; *t *=* *1.4132, df = 8, *P *=* *0.1953)), with means for both populations similarly shifted downward (EF = 369.25 h, SD = 11.15; ZI = 388.89 h, SD = 29.01).

**Figure A6:**
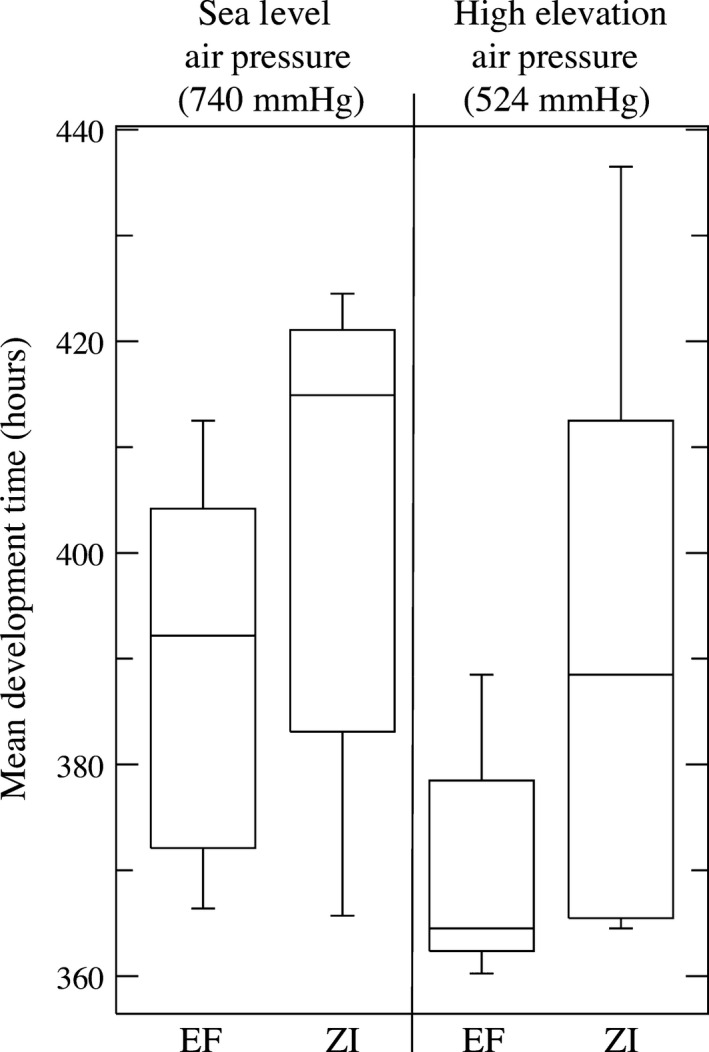
Box plot representation of variation in development time between the highland Ethiopian population (EF) and the lowland Zambian population (ZI) at local air pressure (Madison, WI; 740 mmHg), and at reduced air pressure (524 mmHg) simulating that of Fiche, Ethiopia (3070 m above sea level). Box plots: horizontal line within box, median; box, lower and upper quartiles; capped vertical lines, 95% confidence interval.

Given that larger Ethiopian flies do not result from extended larval development, we tested whether they might instead originate from larger eggs. In agreement with this hypothesis, we found that egg size was significantly larger for EF than for ZI (Fig. 2, right panel; *t *=* *3.4052, df = 16, *P *=* *0.0036). Based on this result, along with the observation of low fecundity in the laboratory, we hypothesized that the larger egg size of Ethiopian flies might come at the expense of laying fewer eggs. Quantifying egg‐laying rate (Fig. 2, center panel), the EF population laid ~3X fewer eggs per 24‐h period on average than ZI (EF = 8.03, SD = 5.08; ZI = 23.72, SD = 6.26), and this difference was statistically significant (*t *=* *0.0024, df = 8, *P *=* *0.0024). To ensure that this result was not attributable to stressed Ethiopian flies at sea level air pressure, we repeated the experiment at the lower native Ethiopian pressure. For both populations, the mean egg‐laying rate was slightly increased under reduced air pressure (EF = 12.75, SD = 7.02; ZI = 31.14, SD = 3.93). The qualitative population difference in egg‐laying rate was therefore unaffected by air pressure (Fig. A6, Table A5), remaining significantly lower for EF (*t *=* *5.11, df = 8, *P *=* *0.0009). Collectively, these results raise the possibility that Ethiopian flies’ large size involves a life history trade‐off favoring female investment in fewer but larger eggs.

### Wing and muscle cell size

We also investigated the cellular mechanisms contributing to the development of larger size in highland Ethiopian flies. In general, tissue size can be enlarged by increases in cell proliferation and/or cell size (Introduction). Cell size often scales with the DNA content of cells; somatic ploidy is a widespread mechanism of developmental size regulation in insects and many other taxa (Edgar and Orr‐Weaver 2001), and it accounts for most growth during *Drosophila* development (Smith and Orr‐Weaver 1991). We therefore tested the contribution of these mechanisms to the size difference between highland and lowland *D. melanogaster* in two different tissues, adult thoracic muscle and adult wing.

**Figure 3:**
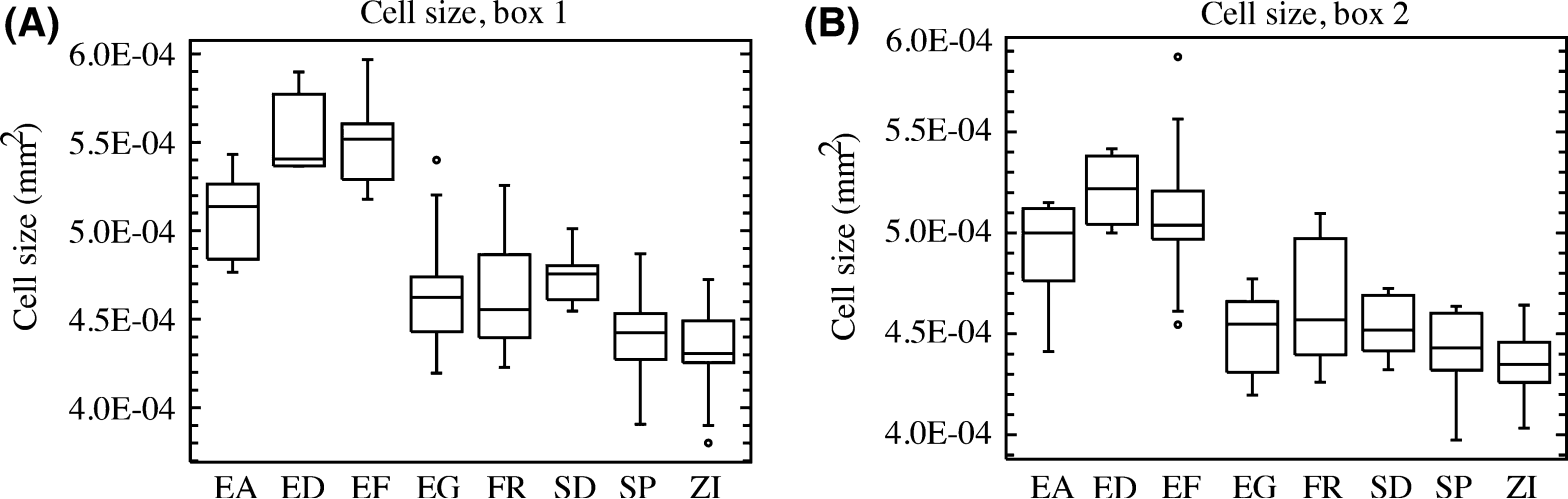
Highland Ethiopian flies (ED/EF) display larger wing cell size. Panels (A) and (B) depict wing cell size from box 1 and box 2, respectively, as illustrated in Figure A3. Box plots are as in Figure 1.

**Figure 4:**
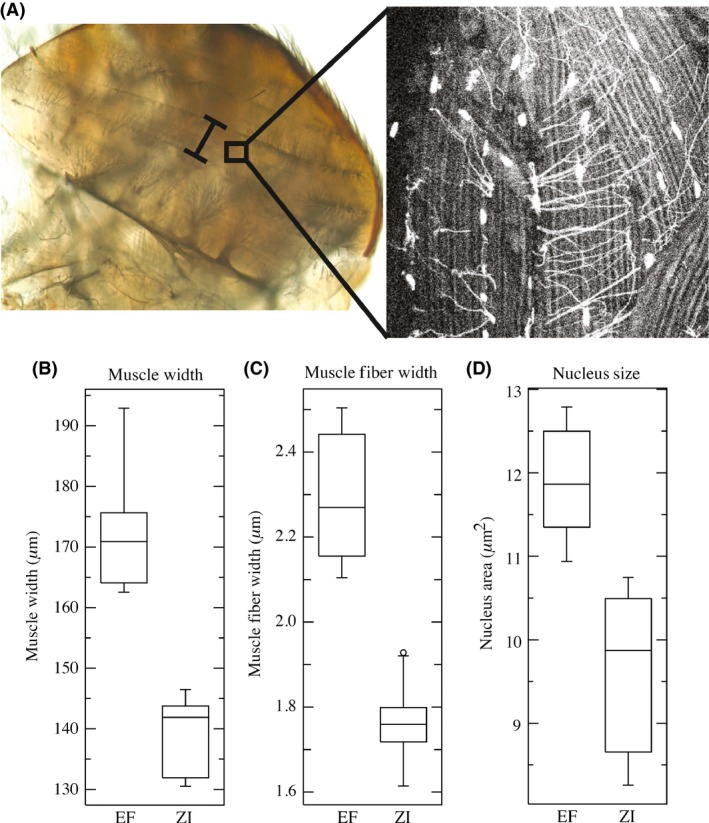
Highland Ethiopian flies show wider thoracic muscles and fibers, and enlarged nuclei. (A) Illustration of the bisected thorax with brackets showing the location and measurement of the third dorsolongitudinal muscle (DLM3). The inset illustrates a representative confocal image of individual muscle fibers and nuclei. (B) Box plot representation of DLM3 width difference between the highland Ethiopian population (EF) and the ancestral range, lowland Zambian population (ZI). (C) Box plot representation of muscle fiber width in the DML3. (D) Box plot representation of muscle cell nucleus size in DLM3 fibers. Box plots are as in Figure 1.

For both wing and skeletal muscle, cells were significantly larger in the highland Ethiopian populations (Figs. 3 and 4). For wing cell size, both of the examined regions returned qualitatively similar patterns across populations, with cells slightly larger in box 1 (Fig. 3). The highland Ethiopian populations both exhibited significantly larger cells than all other populations, all non‐Ethiopian populations exhibited relatively little variation in wing cell size, and the lowland Ethiopian population (EA) was intermediate between these two groups (Fig. 3). Based on population differences (between EF and ZI) in cell size versus wing area, the estimated contribution of cell size to wing size evolution is 42% for box 1, and 29% for box 2. Thus, while cell size varies importantly between these highland and lowland strains, our results imply that cell proliferation may account for a majority of the population wing size difference.

For skeletal muscle (specifically, the dorsal longitudinal muscle of the adult thorax), measurements indicated that cell size variation strongly contributed to the observed increase in thorax size in high‐elevation Ethiopian *D. melanogaster*. Muscle width was significantly larger in the EF population relative to ZI (*t *=* *8.5478, df = 15, *P *=* *0.0001; Fig. 4, left panel), as was muscle fiber width (*t *=* *8.6603, df = 14, *P *=* *0.0001; Fig. 4, center panel). EF also showed larger nucleus size (*t *=* *5.2157, df = 13, *P *=* *0.0002; Fig. 4, right panel), in agreement with the predictions of increased somatic ploidy. Although we cannot measure the size of whole cells for this tissue, the rough similarity of the EF/ZI population ratios observed for whole muscle width (1.24), muscle fiber width (1.29), and nucleus area (1.23) is consistent with a substantial effect of cell size on thoracic size evolution in Ethiopian *D. melanogaster*.

## Discussion

Examples of phenotypic adaptation to high‐altitude environments are pervasive in the literature (reviewed in Keller et al. 2013), due in part to the dramatic variation in environmental factors (e.g., temperature, UV intensity) that occurs over short geographic scales (Heath and Williams 1979; Korner 2007). Size varies along altitudinal gradients for a diversity of animals (Hodkinson 2005; Keller et al. 2013), including drosophilids (Harrison and Carson 1948; Bitner‐Mathe and Klaczko 1999; Dahlgaard et al. 2001; Parkash et al. 2005; Sambucetti et al. 2006; Bridle et al. 2009; Pitchers et al. 2013; Klepsatel et al. 2014; Fabian et al. 2015). Highland Ethiopian *D. melanogaster* constitutes a clear example of the dramatic phenotypes that can arise at high altitude, exhibiting unique features relative to known diversity in *D. melanogaster* (David and Capy 1988), including the largest naturally occurring body and wing size phenotypes described for this species.

Highland Ethiopian thorax and wing length are distinct from all other examined populations. Wing loading has decreased in Ethiopian flies (Table A2, Fig. A4), which could reflect allometric changes with other size traits, or the increased challenge of flight in air that is thinner (Dudley 2000) and colder (Stalker 1980; Josephson 1981; Stevenson and Josephson 1990; Petavy et al. 1997). Further study will be needed to address genetic correlations among size traits that have evolved in Ethiopian *D. melanogaster*, and to examine the influence of potential selective pressures.

As in other *Drosophila* populations and species, Ethiopian *D. melanogaster* shows thermal plasticity for thorax and wing size. While the relative size increase of the highland Ethiopian population is consistent between temperatures in our experiments (Table A4), other factors such as growth medium and larval density may lead to differences between experiments. For example, Pitchers et al. (2013) found a relatively smaller wing size increase for the Ethiopia ED population compared to lowland African samples when grown at 24°C, but a somewhat larger difference at 18°C, while our analysis indicated wing size differences were maximized at 21°C. And compared to our experiments at 20°C, Klepsatel et al. (2014) found a relatively larger contribution of wing cell number versus cell size at 25°C. Hence, further study of the interplay between environmental factors and morphological and cellular size traits is called for.

We failed to detect a significant difference in development time between the Ethiopian highlands and lowland Zambia (Fig. 2). It would be worthwhile to launch a detailed investigation of the interaction between population differences in development time and environmental factors such as rearing temperature. However, our current results do not support the hypothesis larger size in Ethiopian *D. melanogaster* evolved by extending the duration of larval growth.

For egg‐laying rate and egg size, the detected phenotypic differences between the highland EF population and the ancestral ZI population motivate the hypothesis of a trade‐off at high altitude, with increased maternal investment in individual offspring resulting in a lower egg‐laying rate but larger eggs and ultimately larger adults (Fig. 2). Further study is needed to confirm whether these traits constitute a fitness trade‐off in Ethiopian *D. melanogaster*. Work in multiple insect systems suggests that increased egg size can be adaptive at low temperatures, with larger eggs experiencing higher hatch success, higher probability of surviving to adulthood, and shorter larval development times (Fischer et al. 2003a,b; Hassal et al. 2006), but the precise reason for this adaptive advantage remains unclear. For the highland Ethiopian population, the strategy of reduced egg‐laying rate and larger eggs may simply be a by‐product of overall selection to increase adult size without extending developmental time. However, selection may also be acting directly upon egg size to maximize hatching success and embryonic viability (Azevedo et al. 1997). Additional work examining the relationship between adult size, development time, and egg size is needed to tease these explanations apart.

Our cellular analyses provide initial insights into the developmental mechanisms underlying size evolution. Cell proliferation appears to represent a primary contributor to wing size evolution. Cell size plays a role in wings as well and is also a substantial driver of thoracic size evolution (Fig. 4). For body size, the cellular mechanisms driving final adult size in insects are relatively well understood (Brodsky and Uryaeva 1977; Conlon and Raff 1999; Nijhout et al. 2014), and have been implicated in natural variation in body and appendage size (Scholes et al. 2013). Ultimately, adult body size for *D. melanogaster* is determined by the size of the larva when it ceases feeding and enters metamorphosis, and largely by larval muscle size. Demontis and Perrimon (2009) showed that muscle size variation linked to modified insulin/TOR signaling affected adult size. Moreover, they showed that increased size occurred through increasing both nuclei number and DNA content of each individual nucleus (ploidy) through increased endoreplication, in addition to behavioral responses that modulate feeding and nutritional uptake (Conlon and Raff 1999). In the Ethiopian highlands, the individual muscles, muscle cells (fibers), and nuclei were all significantly larger than those of lowland Zambian flies (Fig. 4). Previous studies suggest that nucleus size is an indicator of total DNA content (Maines et al. 2004; Shcherbata et al. 2004; Ohlstein and Spradling 2006). Thus, our results are likely to reflect a role for endoreplication in the evolution of increased body size in the Ethiopian highlands. In addition to being an important developmental regulator of tissue size, somatic ploidy may also be a mechanism of size evolution in insects.

Endoreplication‐mediated cell size differences may contribute to Ethiopian wing size evolution as well. A recent study on laboratory strains found that wing enlargement at cold temperatures was almost entirely driven by larger cells with increased ploidy (Jalal et al. 2015). Modified insulin signaling in the developing wing imaginal disk can inhibit the G2/M transition, prolonging the G1, S, and G2 phases of the cell cycle and increasing wing cell and disk size (Weinkove et al. 1999; Jalal et al. 2015). Thus, it seems plausible that similar mechanisms may be driving shifts in both wing and body size in high‐altitude Ethiopian *D. melanogaster*.

Ultimately, the data we present illustrate the complex nature of body size evolution in high‐elevation Ethiopian *D. melanogaster*, with this population undergoing changes in reproductive strategy and the cell cycle. While this analysis does not directly test for a link between each of these individual phenotypes and the overarching size phenotype, past work suggests their likely interconnection (Parsons 1962; Harvey 1983, 1985; Partridge et al. 1994a; James and Partridge 1995; Azevedo et al. 1997; Conlon and Raff 1999). Moreover, understanding developmental and cellular level processes underlying phenotypic variation can provide insight into the genetic underpinnings of trait evolution. For complex traits, this can be exceptionally useful in understanding how causative genetic loci have been manipulated in the context of networks of interacting genes, developmentally correlated traits (e.g., body and appendage size), and organismal physiology.

## Conflict of Interest

None declared.

## Data Archiving

All data will be available at: http://dx.doi.org/10.5061/dryad.mp8rt.

## Appendix

**Table A1:** Geographic and collection information for the populations used in this study

Pop ID	Country	Locality	Latitude	Longitude	Elev. (m)	Collect Date	Collector
EA	Ethiopia	Gambella	8.25	34.59	525	12/2011	R. Corbett‐Detig
ED	Ethiopia	Dodola	6.98	39.18	2492	12/2008	J. Pool
EF	Ethiopia	Fiche	9.81	38.63	3070	12/2011	R. Corbett‐Detig
EG	Egypt	Cairo	30.10	31.32	25	1/2011	R. Corbett‐Detig
FR	France	Lyon	45.77	4.86	175	7/2010	J. Pool
SD	South Africa	Dullstroom	−25.42	30.10	2000	12/2011	J. Pool
SP	South Africa	Phalaborwa	−23.94	31.14	350	7/2010	R. Corbett‐Detig
ZI	Zambia	Siavonga	−16.54	28.72	530	7/2010	R. Corbett‐Detig

**Table A2:** Wing and thorax length means and standard deviations for each analyzed population, and the ratio of wing length to thorax length (WL/TL)

Pop.	Number of inbred lines	Mean wing length (WL)	SD	Mean wing depth (WD)	SD	WL/TL
EA	30	1.370	0.0500	1.294	0.0322	1.059
ED	6	1.508	0.0350	1.345	0.0333	1.121
EF	31	1.532	0.0310	1.345	0.0194	1.135
EG	20	1.326	0.0240	1.241	0.0218	1.068
FR	83	1.411	0.0295	1.262	0.0228	1.117
SD	70	1.326	0.0413	1.239	0.0295	1.070
SP	10	1.297	0.0273	1.230	0.0238	1.054
ZI	52	1.282	0.0375	1.226	0.0279	1.046

**Table A3:** Mean values for each of five crosses from the highland Ethiopian population (EF) and lowland Zambian population (ZI) for wing area (mm^2^), mass per female (mg), and wing loading (mg/mm^2^). Wing area values were averaged across 5 F1 females from each cross, and per‐female mass was calculated by weighing 20 F1 females from each cross

Cross	Wing area	Mass	Wing loading
EF39N × EF83N	2.932	1.44	0.491
EF117N × EF81N	2.729	1.46	0.534
EF32N × EF78N	2.978	1.66	0.557
EF54N × EF93N	2.930	1.61	0.549
EF59N × EF95N	2.766	1.53	0.553
ZI125N × ZI323N	1.751	1.17	0.668
ZI160N × ZI455N	1.773	1.17	0.660
ZI186N × ZI315N	1.585	1.21	0.763
ZI216N × ZI482N	1.852	1.35	0.729
ZI206N × ZI485N	1.827	1.19	0.651

**Table A4:** Comparisons of wing length (WL), thorax length (TL), and WL/TL for a single high‐altitude Ethiopian line (EF98) and a single low‐altitude Zambian line (ZI18) at a range of developmental temperatures

Dev. Temp. (°C)	EF98			ZI18		
	Wing length (mm)	Thorax length (mm)	WL/TL	Wing length (mm)	Thorax length (mm)	WL/TL
14	3.14	1.106	2.84	2.652	1.002	2.65
17	3.18	1.140	2.79	2.696	1.056	2.55
21	3.12	1.148	2.72	2.500	1.034	2.42
25	2.97	1.106	2.69	2.400	1.036	2.36
28	2.86	1.092	2.62	2.296	0.986	2.33
31	2.60	0.992	2.58	2.164	0.904	2.39

**Table A5:** Comparisons of egg‐laying rate and development time between highland Ethiopia (EF) and lowland Zambia (ZI) at the local air pressure (Madison, WI; 740 mmHg) and simulated high‐elevation air pressure of 3000 m elevation (524 mmHg) for outcrossed (F1) female *D. melanogaster*. Each value is the average across >10 individuals per cross

Line 1	Line 2	Egg‐laying rate (eggs/female/day)		Development Time (hours)	
		Low‐altitude pressure	High‐altitude pressure	Low‐altitude pressure	High‐altitude pressure
EF8N	EF26N	15.14	17.00	395.83	403.05
EF6N	EF43N	4.67	1.88	392.18	387.00
EF10N	EF39N	1.88	12.00	377.83	360.25
EF15N	EF32N	9.78	12.38	366.38	363.08
EF19N	EF27N	8.67	20.50	412.50	376.50
ZI192N	ZI125N	32.67	24.60	365.73	390.35
ZI337N	ZI160N	16.56	32.43	417.64	386.79
ZI278N	ZI159N	24.13	31.00	400.50	370.44
ZI256N	ZI134N	26.00	32.67	414.90	412.50
ZI197N	ZI124N	19.25	35.00	424.50	369.38
